# Affektive Störungen: Entwicklungen der ICD-11 im Vergleich zur ICD-10

**DOI:** 10.1007/s00115-025-01874-y

**Published:** 2025-08-20

**Authors:** Martin Härter, Frank Schneider

**Affiliations:** 1 Zentrum für Psychosoziale Medizin, Institut und Poliklinik für Medizinische Psychologie und Institut für Psychotherapie (IfP), Universitätsklinikum Hamburg-Eppendorf, Martinistraße 52, 20246 Hamburg, Deutschland; 2 Institut für Geschichte, Theorie und Ethik der Medizin, Heinrich-Heine-Universität Düsseldorf, Düsseldorf, Deutschland

**Keywords:** Depressive Störung, Bipolare Störung, Cluster, Klassifikation, ICD-11, Depressive disorder, Bipolar disorder, Cluster, Classification, ICD-11

## Abstract

Mit Einführung der 11. Revision der Internationalen statistischen Klassifikation der Krankheiten und verwandter Gesundheitsprobleme (ICD-11) der World Health Organization wurden strukturelle und inhaltliche Anpassungen der diagnostischen Richtlinien für affektive Störungen vorgenommen, die in diesem Übersichtsartikel dargestellt werden. Durch die Aktualisierung ergeben sich einige Änderungen bez. der diagnostischen Einteilung der affektiven Störungen, angelehnt an das amerikanische Diagnostic and Statistical Manual of Mental Disorders 5 (DSM-5). Die ICD-11 ordnet depressive Symptome sog. Clustern zu, die Hauptsymptome depressive Stimmung und Freudlosigkeit können von kognitiven, verhaltensbezogenen oder neurovegetativen Symptomen begleitet sein. Eine persistierende depressive Störung liegt vor, wenn die depressive Episode ununterbrochen mehr als 2 Jahre andauert. Eine bipolare Störung wird künftig in Typ I und Typ II unterschieden. Manische Episoden sind weiterhin nur kodierbar im Rahmen bipolarer Störungen und können nicht als eigenständige, separate Störung diagnostiziert werden. Das Konzept der anhaltenden affektiven Störungen in der ICD-10 wird verlassen, Dysthymie wird den depressiven Störungen und Zyklothymie den bipolaren Störungen zugeordnet.

## Abstract (en)

With the introduction of the 11th revision of the World Health Organization (WHO) “International Statistical Classification of Diseases and Related Health Problems” (ICD-11), structural and content-related adjustments were made to the diagnostic guidelines for affective disorders, which are presented in this review article. The update has resulted in some changes to the diagnostic classification of affective disorders, based on the American Diagnostic and Statistical Manual of Mental Disorders 5 (DSM-5). The ICD-11 assigns depressive symptoms to so-called clusters, the main symptoms of depressed mood and joylessness can be accompanied by cognitive, behavioral or neurovegetative symptoms. In the case of remission of depressive episodes, the ICD-11 distinguishes between partial and complete remission. A persistent depressive disorder is present if the depressive episode lasts continuously for more than 2 years. In future, bipolar disorder will be divided into type I and type II. Manic episodes can still only be coded in the context of bipolar disorders and cannot be diagnosed as an independent, separate disorder. The concept of persistent affective disorders in the ICD-10 is abandoned, dysthymia is categorized as a depressive disorder and cyclothymia as a bipolar disorder.

## Hintergrund

Affektive Störungen sind eine Gruppe von Erkrankungen, deren Hauptmerkmale eine zumeist phasenhaft ausgeprägte Veränderung der Stimmung, hin zum depressiven oder manischen Pol, und eine Änderung der Antriebslage sind. Von den unipolaren depressiven Verläufen sind die bipolaren affektiven Störungen zu unterscheiden, bei denen sowohl hypomanische oder manische als auch depressive oder gemischte Episoden mit mehr oder weniger symptomfreien Intervallen vorkommen.

Ziel des Beitrags ist eine komprimierte übersichtliche Darstellung der Veränderungen der Klassifikation affektiver Störungen im Übergang von der International Statistical Classification of Diseases and Related Health Problems 10 (ICD-10) auf die ICD-11. Einerseits wird die taxonomische Gliederung der depressiven und bipolaren Störungen tabellarisch dargestellt, andererseits werden spezifische Hinweise zur Diagnosestellung bez. depressiver Störungen gegeben. Für detailliertere Informationen wird auf die entsprechenden Klassifikationssysteme der umfassenden Erkrankungskategorien inklusive ihrer Spezifika verwiesen, einsehbar über das Bundesinstitut für Arzneimittel und Medizinprodukte^1^^,^^2^.

## Veränderungen der Taxonomie affektiver Störungen im Übergang von ICD-10 zu ICD-11

Strukturell ergaben sich im Übergang von ICD-10 zu ICD-11 folgende Änderungen [siehe auch 1–7]:

Neben der Einteilung in *unipolare und bipolare Verläufe* wurden in der ICD-10 bislang die episodenhaften affektiven Störungen, d. h. *manische *(Kapitel F 30–31) oder *depressive Episoden* (Kapitel F 32–33), von *chronisch anhaltenden affektiven Störungen* (Dysthymie, Zyklothymie) abgegrenzt (Kapitel F. 34). Darüber hinaus bestanden die Restkategorien „andere affektive Störungen“ (Kapitel F 38) bzw. „nicht näher bezeichnete affektive Störung“ (Kapitel F 39).

Die Diagnostik affektiver Störungen bleibt in der ICD-11 im Prinzip *kategorial*, jedoch ist das Ausmaß der Operationalisierung (also Zählen von Symptomen) zugunsten einer klinischen Beurteilung mit den zentralen Kriterien Symptomausprägung und Funktionseinbußen zurückgenommen worden. Das Konzept der anhaltenden affektiven Störungen des ICD-10 wird im ICD-11 verlassen, *Dysthymie *wird den *depressiven Störungen *und *Zyklothymie *den *bipolaren Störungen* zugeordnet.

**Abb. 1:**
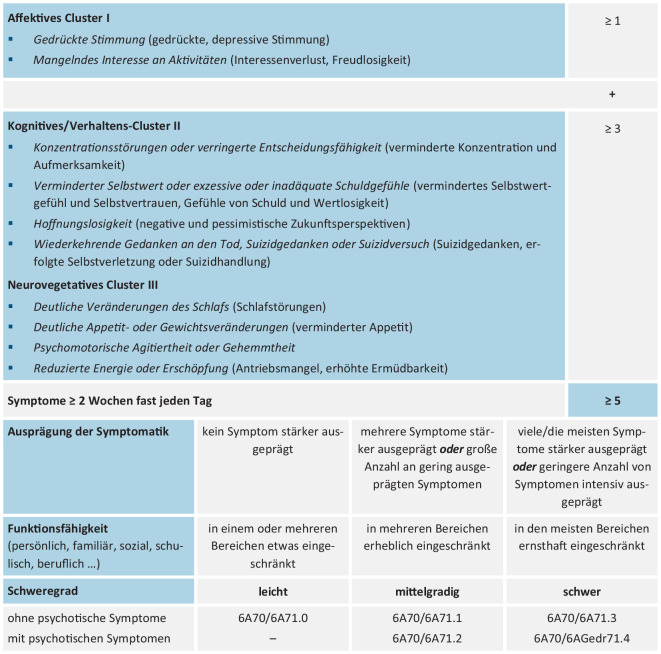
Diagnostische Leitlinien (ICD-11): Depressive Störung mit Einzelepisode oder rezidivierende depressive Störung (Symptome/Kriterien der ICD-10 in Klammer.) *ICD* International Statistical Classification of Diseases and Related Health Problems

In der ICD-11 werden die *Differenzierung der depressiven Symptomatik* in drei Symptomcluster, eine dimensionale Beurteilung des Schweregrads und größere Freiheitsgrade für klinisch tätiges Fachpersonal in der Diagnostik affektiver Störungen verwirklicht (Abb. 1). Zusätzlich besteht die Zusatzkodierung „mit psychotischen Symptomen“ auch bei mittelschwerer depressiver Episode. Bei gegenwärtig remittierten depressiven Episoden unterscheidet die ICD-11 darüber hinaus zwischen einer *partiellen* und *vollständigen Remission*.

Als neue Kategorien werden die „*prämenstruelle dysphorische Störung*“ und „*gemischte depressive Störung und Angststörung*“ aufgenommen.

Nach Erstmanifestation einer depressiven Episode treten bei der Mehrzahl der Patienten (55–65 %) im Laufe des Lebens mehrere depressive Phasen auf (*rezidivierende depressive Störung*). Mit jeder weiteren depressiven Episode steigt das Wiedererkrankungsrisiko. Zudem verkürzen sich mit steigender Anzahl der Episoden ebenso wie mit zunehmendem Lebensalter die zeitlichen Abstände zwischen den depressiven Phasen (s. Taxonomie der rezidivierenden Depression [ICD-10] bzw. der rezidivierenden depressiven Störung [ICD-11], Tab. 1).

**Tab. 1:** Taxonomie der depressiven Störungen im Vergleich von ICD-10 und ICD-11

ICD-10	ICD-11 (Entwurfsfassung)
*Affektive Störungen* *(F30–31)*	*Affektive Störungen* *(6A60.X – 6A73.X)*
*F 32.– Depressive Episode*	*6A70 Depressive Störung mit Einzelepisoden*
F32.0 Leichte depressive Episode	6A70.0 Depressive Störung mit Einzelepisode, leichtgradig
F32.1 Mittelgradige depressive Episode	6A70.1 Depressive Störung mit Einzelepisode, mittelgradig, ohne psychotische Symptome 6A70.2 Depressive Störung mit Einzelepisode, mittelgradig, mit psychotischen Symptomen
F32.2 Schwere depressive Episode ohne psychotische Symptome	6A70.3 Depressive Störung mit Einzelepisode, schwergradig, ohne psychotische Symptome
F32.3 Depressive Störung, gegenwärtig schwere Episode mit psychotischen Symptomen	6A70.4 Depressive Störung mit Einzelepisode, schwergradig, mit psychotischen Symptomen
F32.4 Depressive Störung, gegenwärtig remittiert	6A70.6 Depressive Störung mit Einzelepisode, gegenwärtig in Teilremission 6A70.7 Depressive Störung mit Einzelepisode, gegenwärtig in Vollremission
F32.8 Sonstige depressive Episoden Atypische Depression	6A8Y Sonstige näher bezeichnete affektive Störungen
F32.9 Depressive Episode, nicht näher bezeichnet	6A8Z Affektive Störungen, nicht näher bezeichnet
*F33.– Rezidivierende Depression*	*6A71 Rezidivierende depressive Störung*
F33.0 Rezidivierende depressive Störung, gegenwärtig leichte Episode	6A71.0 Rezidivierende depressive Störung, gegenwärtig leichtgradige Episode
F33.1 Rezidivierende depressive Störung, gegenwärtig mittelgradige Episode	6A71.1 Rezidivierende depressive Störung, gegenwärtig mittelgradige Episode, ohne psychotische Symptome 6A71.2 Rezidivierende depressive Störung, gegenwärtig mittelgradige Episode, mit psychotischen Symptomen
F33.3 Rezidivierende depressive Störung, gegenwärtig schwere Episode ohne psychotische Symptome	6A71.3 Rezidivierende depressive Störung, gegenwärtig schwergradige Episode, ohne psychotische Symptome
F33.3 Rezidivierende depressive Störung, gegenwärtig schwere Episode mit psychotischen Symptomen	6A71.4 Rezidivierende depressive Störung, gegenwärtig schwergradige Episode, mit psychotischen Symptomen
F33.4 Rezidivierende depressive Störung, gegenwärtig remittiert	6A71.6 Rezidivierende depressive Störung, gegenwärtig in Teilremission 6A71.7 Rezidivierende depressive Störung, gegenwärtig in Vollremission
F33.8 Sonstige rezidivierende depressive Störungen	6A71.Y Sonstige näher bezeichnete rezidivierende depressive Störung
F33.9 Rezidivierende depressive Störung, nicht näher bezeichnet	6A71.Z Rezidivierende depressive Störung, nicht näher bezeichnet
*F34.– Anhaltende affektive Störungen*	*–*
F34.1 Dysthymia	6A72 Dysthyme Störung
F34.8 Sonstige anhaltende affektive Störungen	–
F34.8 Anhaltende affektive Störung, nicht näher bezeichnet	–
*Neue Kategorien*	–
F41.2 Angst und depressive Störung, gemischt	6A73 Gemischte depressive Störung und Angststörung
–	GA34.41 Prämenstruelle dysphorische Störung

*ICD *International Statistical Classification of Diseases and Related Health Problems

Die *bipolare Störung* wird künftig in *Typ I* und *Typ II* unterschieden. Manische Episoden sind weiterhin nur kodierbar im Rahmen bipolarer Störungen und können nicht als eigenständige, separate Störung diagnostiziert werden (Tab. 2).

**Tab. 2:** Taxonomie der bipolaren Störungen im Vergleich von ICD-10 und ICD-11

ICD-10	ICD-11 (Entwurfsfassung)
*Affektive Störungen* *(F30–39)*	*Affektive Störungen* *(6A60.X – 6A61.X)*
*F 30.– Manische Episode*	*6A60 Bipolare Störung Typ I*
F30.0 Hypomanie	6A60.2 (oder 6A61.2) Bipolare Störung Typ I (oder Typ II), gegenwärtig hypomanische Episode
F30.1 Manie ohne psychotische Symptome	6A60.0 Bipolare Störung Typ I, gegenwärtig manische Episode, ohne psychotische Symptom
F30.2 Manie mit psychotischen Symptomen	6A60.1 Bipolare Störung Typ I, gegenwärtig manische Episode, mit psychotischen Symptomen
F30.8 Sonstige manische Episoden	6A8Y Sonstige näher bezeichnete affektive Störungen
F30.9 Manische Episode, nicht näher bezeichnet	6A8Z Affektive Störungen, nicht näher bezeichnet
*F31.– Bipolare affektive Störung*	*6A60 Bipolare Störung Typ I*
F31.0 Bipolare affektive Störung, gegenwärtig hypomanische Episode	6A60.2 (oder 6A61.2) Bipolare Störung Typ I (oder Typ II), gegenwärtig hypomanische Episode
F31.1 Bipolare affektive Störung, gegenwärtig manische Episode ohne psychotische Symptome	6A60.0 Bipolare Störung Typ I, gegenwärtig manische Episode, ohne psychotische Symptom
F31.2 Bipolare affektive Störung, gegenwärtig manische Episode mit psychotischen Symptomen	6A60.1 Bipolare Störung Typ I, gegenwärtig manische Episode, mit psychotischen Symptomen
F31.3 Bipolare affektive Störung, gegenwärtig leichte oder mittelgradige depressive Episode	6A60.3 Bipolare Störung Typ I, gegenwärtig leichtgradige depressive Episode 6A60.4 Bipolare Störung Typ I, gegenwärtig mittelgradige depressive Episode, ohne psychotische Symptome
F31.4 Bipolare affektive Störung, gegenwärtig schwere depressive Episode ohne psychotische Symptome	6A60.6 Bipolare Störung Typ I, gegenwärtig schwergradige depressive Episode, ohne psychotische Symptome
F31.5 Bipolare affektive Störung, gegenwärtig schwere depressive Episode mit psychotischen Symptomen	6A60.7 Bipolare Störung Typ I, gegenwärtig schwergradige depressive Episode, mit psychotischen Symptomen
F31.6 Bipolare affektive Störung, gegenwärtig gemischte Episode	6A60.9 Bipolare Störung Typ I, gegenwärtig gemischte Episode, ohne psychotische Symptome 6A60.A Bipolare Störung Typ I, gegenwärtig gemischte Episode, mit psychotischen Symptomen
F31.7 Bipolare affektive Störung, gegenwärtig remittiert	6A60.F Bipolare Störung Typ I, gegenwärtig in Vollremission
F31.8 Sonstige bipolare affektive Störungen	6A60.Y Sonstige näher bezeichnete bipolare Störung Typ I
F31.9 Bipolare affektive Störung, nicht näher bezeichnet	6A60.Z Bipolare Störung Typ I, nicht näher bezeichnet
–	*6A61 Bipolare Störung Typ II*
–	6A61.0 Bipolare Störung Typ II, gegenwärtig hypomanische Episode
–	6A61.1 Bipolare Störung Typ II, gegenwärtig leichtgradige depressive Episode
–	6A61.2 Bipolare Störung Typ II, gegenwärtig mittelgradige depressive Episode, ohne psychotische Symptome
–	Usw.
*F34.– Anhaltende affektive Störungen*	–
F34.0 Zyklothymia	6A62 Zyklothyme Störung
F34.8 Sonstige anhaltende affektive Störungen	–
F34.9 Anhaltende affektive Störung, nicht näher bezeichnet	–

*ICD *International Statistical Classification of Diseases and Related Health Problems,* Usw.* Kodierungen weiter wie bei Bipolare Störung Typ I bis 6A61.Z Bipolare Störung Typ II, nicht näher bezeichnet

Zur Erfassung interindividueller Unterschiede können depressive oder manische Episoden mit sog. „Qualifiern“ (6A80*) zusatzkodiert werden (analog der „Specifier“ des Diagnostic and Statistical Manual of Mental Disorders 5 [DSM-5]), z. B. bez. begleitender Angstsyndrome, eines chronischen Verlaufs, eines saisonalen Musters oder in Bezug auf die Peripartalzeit [4]. Es gibt es in der ICD-11 die Möglichkeit, zusätzlich zur affektiven Stammdiagnose „Depression“ oder „bipolare Störung“ weitere klinische Erscheinungsformen zu kodieren, z. B. „mit ausgeprägten Angstsymptomen“ (6A80.0); „mit Panikattacken“ (6A80.1); „mit anhaltender Symptomatik“, d. h. mind. 2 Jahre (= Möglichkeit, chronische Depression, die über eine Dysthymie hinausgeht, zu kodieren; 6A80.2); „mit Melancholie“ (6A80.3, was in der ICD-10 als „somatisches Syndrom“ kodiert wurde); „mit saisonalem Beginn“ (6A80.4) oder „mit rapid cycling“ (6A80.5).

## Diagnose depressiver Episoden

Für die Diagnose einer depressiven Episode müssen *mindestens 5 Symptome* (in der ICD-10 genügten 4) vorliegen, davon *mindestens eines aus dem affektiven Cluster*. Die Einstufung der Episodenschwere (leicht, mittelgradig, schwer) erfolgt – anders als in der ICD-10 – nicht anhand der Summe der Symptome, sondern berücksichtigt neben der Anzahl auch deren *Intensität *sowie den *Grad der Funktionseinschränkung*. Die Gruppierung der depressiven Symptome soll die große klinische Breite der Symptomatologie verdeutlichen und erhöht die Übersichtlichkeit. Im Cluster I finden sich die beiden sog. „Eingangssymptome“ („entry level symptoms“). Nicht mehr dazu zählt das weniger spezifische Symptom „Antriebsmangel, erhöhte Ermüdbarkeit“, welches nun als „reduzierte Energie oder Erschöpfung“ zum Cluster III gehört.

## Schlussbetrachtung

Es ist zu hoffen, dass die neue ICD-11 nach ihrer Einführung in die Versorgung in Deutschland auf große Akzeptanz stoßen wird. Zwar wird die Kodierung differenzierter und gegebenenfalls kleinteiliger, dies entspricht aber auch dem allgemeinen Bemühen um eine noch stärkere Personalisierung bezüglich einzusetzender Behandlungsstrategien. Die stärkere Betonung der Dimensionalität von Störungen, z. B. im Hinblick auf die Funktionsbeeinträchtigung und den Schweregrad, können dazu beitragen, dass neben der klinischen Reduktion von Symptomen vor allem auch die Verbesserung der Funktionseinschränkungen und der Lebensqualität als therapeutische Ziele mehr Bedeutung gewinnen.

## Fazit für die Praxis

- Bei den in der Allgemeinbevölkerung sehr häufigen vorübergehenden depressiven Verstimmungen ist die Grenzziehung zu depressiven Störungen mithilfe der Beurteilung des Schweregrades der Symptome und der Funktionseinschränkungen sinnvoll.
- Die Gruppierung bzw. Clusterung depressiver Symptome ist aufgrund der klinischen Breite der Symptomatologie sinnvoll und erhöht die Übersichtlichkeit.
- Die Ergänzung der persistierenden oder chronischen Depression war überfällig und wurde bereits im klinischen Alltag häufig verwendet.
- Die Ergänzung der Klassifikation durch die „Qualifier“ ist klinisch sinnvoll, da zahlreiche Symptome wie Angst, Panik, Persistenz, saisonales Muster oder Melancholie häufig sind und für die Behandlungsplanung sinnvoll sind.
- Das Aufgeben der Kodierung einzelner manischer Episoden ist folgerichtig, da die Trennung im Rahmen von Familien‑, Therapie- und Verlaufsstudien nicht gut belegbar war.
- Die eigenständige Kodierung der bipolaren Störung Typ II ist begrüßenswert, da Studienerkenntnisse diese Sichtweise stützen und die Prognose bzw. das Ansprechen auf Therapien meist günstiger verlaufen.
- Die Auflösung der anhaltenden affektiven Störungen und die Zuordnung in die jeweiligen Kapitel depressive bzw. bipolare Störungen ist vor allem wegen der ähnlichen Behandlungsstrategien hilfreich, darüber hinaus zeigen sich häufig klinische Verläufe mit Übergängen bei diesen Subkategorien.

## Footnotes

1 BfArM – ICD-10-GM Version 2024 (https://klassifikationen.bfarm.de/icd-10-gm/kode-suche/htmlgm2024/block-f30-f39.htm).

2 BfArM – ICD-11 in Deutsch – Entwurfsfassung (https://www.bfarm.de/DE/Kodiersysteme/Klassifikationen/ICD/ICD-11/uebersetzung/_node.html).
